# Oral Potentially Malignant Disorders and *Candida* in Oral Tongue Squamous Cell Carcinoma Patients

**DOI:** 10.3390/dj11070170

**Published:** 2023-07-14

**Authors:** Orvokki Saraneva, Jussi Furuholm, Jaana Hagström, Timo Sorsa, Ville Rita, Taina Tervahartiala, Hannamari Välimaa, Hellevi Ruokonen

**Affiliations:** 1 Department of Oral and Maxillofacial Diseases, University of Helsinki, FI-00290 Helsinki, Finland; 2 Department of Oral and Maxillofacial Diseases, Helsinki University Hospital and University of Helsinki, FI-00290 Helsinki, Finland; 3 Department of Pathology, University of Helsinki and Helsinki University Hospital, FI-00014 Helsinki, Finland; 4 Department of Oral Pathology and Radiology, University of Turku, FI-20520 Turku, Finland; 5 Division of Periodontology, Department of Dental Medicine, Karolinska Institute, SE-17177 Stockholm, Sweden; 6 Department of Virology, University of Helsinki, FI-00290 Helsinki, Finland; 7 Meilahti Infectious Diseases and Vaccine Research Center, MeVac, Helsinki University Hospital and University of Helsinki, FI-00290 Helsinki, Finland

**Keywords:** oral tongue squamous cell carcinoma, *Candida*, lichen planus, lichenoid reaction

## Abstract

This retrospective study addressed the role of oral potentially malignant disorders and the presence of intraepithelial *Candida* hyphae in the carcinogenesis of the oral tongue squamous cell carcinoma and its association with smoking, alcohol consumption, and oral inflammatory burden. The medical records of 183 subjects diagnosed with oral tongue squamous cell carcinoma at the Helsinki University Hospital were investigated. Preceding oral lichen planus, lichenoid reaction, and leukoplakia diagnosis were recorded. Further, the data on *Candida* hyphae in histological samples as an indicator of oral candidiasis, oral inflammatory burden, smoking, and alcohol consumption were recorded and analyzed. The histopathological diagnosis of oral lichen planus/lichenoid reaction (*p* < 0.001) and the presence of *Candida* hyphae (*p* = 0.005) were associated significantly with female gender. Oral lichen planus/lichenoid reaction patients were less often smokers than patients without these lesions. *Candida* hyphae were more often recorded in patients without alcohol use (*p* = 0.012). Oral lichen planus/lichenoid reaction and *Candida* hyphae in histological samples were associated with female gender and lower levels of typical risk factors, such as alcohol use and smoking, in oral tongue squamous cell carcinoma patients. Therefore, these patients should be well monitored despite a potential lack of the classical risk factors of oral carcinoma.

## 1. Introduction

Oral potentially malignant disorders (OPMDs) include leukoplakia, erythroplakia, erythroleukoplakia, proliferative verrucous leukoplakia, oral lichen planus, oral submucous fibrosis, palatal lesions in reverse smokers, smokeless tobacco keratosis, oral lupus erythematosus, actinic keratosis, dyskeratosis congenita, oral lichenoid lesion, oral graft versus host disease, syphilic glossitis, and chronic candidiasis [1,2,3,4]. OPMD conditions have an increased risk of progressing into malignancy, but the risk varies individually [5,6,7].

The composition of oral microbiota may be one factor contributing to the malignant transformation of premalignant lesions to oral squamous cell carcinoma (OSCC) [8,9,10,11]. In particular, changes in the relative abundance of certain oral microbes such as *Candida* species, *Porphyromonas gingivalis*, *Fusobacterium nucleatum*, and *Streptococcus* species have been shown to associate with OSCC [12].

Mucosal barrier abnormalities and the use of immunosuppressive medication, both typical for OLP, predispose to candidiasis [13,14]. Indeed, oral *Candida* infections are observed more commonly in patients with oral lichen planus (OLP) than patients without OLP [15,16]. Non-*Candida albicans* (*C. albicans*) species, particularly *C. glabrata* and *C. krusei*, have been found more often in patients with erosive OLP, while *C. albicans* has been more frequently observed in patients with non-erosive OLP [15,16,17].

*Candida* species and their biotypes are believed to have a role in oral carcinogenesis [15,18,19]. *Candida* isolates from oral tongue squamous cell carcinoma (OTSCC) patients have been shown to produce more virulence factors than isolates from non-OTSCC patients [20]. *Candida* proteases can activate latent proMMP-8 and promote tissue destruction [21]. The virulence factors of *Candida* vary according to the oral mucosal lesion type [20]. In erosive and non-erosive OLP patients, *Candida* phospholipase enzyme activity is higher than in the controls [17] and *Candida* species and biotypes differ between these OLP lesions as well as between homogeneous and non-homogeneous leukoplakia lesions [19]. Non-homogeneous leukoplakia is believed to develop more often into oral cancer than homogeneous leukoplakia [19,22]. *C. albicans* has been shown to have different biotypes in non-homogeneous leukoplakia, suggesting that it might play a role in carcinogenesis [19].

A correlation between the prevalence of *Candida* colonization of oral mucosal lesions and the severity of epithelial dysplasia has been reported [23,24]. It is unclear whether colonization is a consequence of oral epithelial changes or precedes them. The tongue lesions are more often infected with *Candida* species than lesions in other parts of the oral cavity [23,24]. OSCC patients are more frequently colonized with *Candida* species than controls [25]. *Candida* species from OSCC patients have a greater ability to form biofilm than those from non-OSCC patients [26]. In addition, *Candida* biofilms from OSCC patients produce more carcinogenic acetaldehyde and have higher metabolic activity than biofilms from non-oral cancer [26]. *C. parapsilosis* and *C. albicans* genotype B is significantly more common in non-oral cancer patients, whereas *C. albicans* genotype A is more frequently isolated in oral cancer patients, indicating that genotypic differences in *Candida* species may have a role in carcinogenesis [25].

*C. albicans* has been shown to produce carcinogens including nitrosamines, which can activate proto-oncogenes triggering carcinomatous changes [27]. Furthermore, candidal proteases in concert with proteases from oral dysbiotic periodontopathogens can activate host cell-derived latent pro matrix metalloproteases in tissue destruction cascades, which is important in the development and etiopathogenesis of oral malignancies [21,28,29].

Previous studies have shown that *C. albicans* isolates in potentially carcinogenic oral lesions can produce mutagenic levels of acetaldehydes and tissue destructive proteases [21]. Smoking and alcohol abuse may favor adaptive changes, resulting in the upregulation of *Candida* acetaldehyde metabolism [30]. Therefore, all *Candida* species may produce potentially carcinogenic amounts of acetaldehyde, especially in combination with smoking or alcohol consumption [26]. Acetaldehyde production is known to vary among species, and *C. albicans*, *C. tropicalis*, and *C. parapsilosis* produce more acetaldehyde than other species in the same genus [31].

*Candida* species can produce mutagenic amounts of acetaldehyde and tissue destructive proteases in mucosal lesions [21]. Furthermore, it is recognized that smoking and alcohol use may favor adaptive changes, resulting in re-regulation of *Candida* acetaldehyde metabolism and accelerated acetaldehyde production [26,30].

Oral polymicrobial interactions may further favor oral colonization and biofilm formation as well as modify inflammatory reactions by the *Candida* species [32]. With this background, the primary aim of this retrospective observational study was to clarify the role of intraepithelial *Candida* hyphae finding, indicative of *Candida* infection, in association with certain OPMD lesions in OTSCC carcinogenesis. The secondary objective was to evaluate the effect of dental health, alcohol consumption, and smoking as contributing factors—both independently and in conjunction with the primary factors. The nature of the study was retrospective, collecting information from the patient files and pathology reports.

## 2. Materials and Methods

### 2.1. Study Design

We studied retrospectively the medical records of 183 consecutive patients with OTSCC treated in 2016–2017 at the Department of Oral Maxillofacial Diseases, Helsinki University Hospital, Finland. The patients were identified from the hospital electronic medical records using the International Classification of Diseases, Tenth Revision (ICD-10) codes C01, C02.0, C02.1, C02.11, C02.2, and C02.3. All patients identified with these codes and confirmed to have OTSCC based on the medical records were included in the study. A lack of electronic medical records or electronic histopathological report was used as the exclusion criteria. No informed consent was required due to the retrospective nature of the study.

Information on OTSCC, concurrent systemic autoimmune diseases, immunosuppressive medications, alcohol consumption, smoking, clinical oral health status, and panoramic tomography findings were gathered from the medical records. Any previous diagnosis of oral leukoplakia, OLP, and lichenoid reaction (LR) and the presence of oral *Candida* hyphae were ascertained from histopathological reports. Histopathological samples were taken from some patients with OPMD years before oral cancer diagnoses and some patients’ first samples from cancer lesions. The follow-up time ranged from 11 years to 0 years.

All oral mucosal tissue samples had been processed with hematoxylin and eosin staining and were investigated further by Periodic acid-Schiff (PAS) staining in order to visualize *Candida* hyphae. We used the World Health Organization’s diagnostic criteria for the determination of LR and OLP [33,34]. Due to a lack of precise differential diagnostic information on clinical manifestation and the retrospective nature of the study, patients with LR and/or OLP were analyzed as one group. Accordingly, we could not evaluate the presence of possible clinical findings typical for candidiasis. *Candida* culture results were only available from very few patients. Therefore, we were unable to acquire all the necessary information to meet all the criteria for *Candida* infection [35]. Instead, the detection of intraepithelial *Candida* hyphae by PAS staining was used as the criterion of active candidiasis.

Dental health and oral infection burden were evaluated using the following parameters: Periodontal Inflammatory Burden Index (PIBI), describing the inflammatory load caused by periodontitis, Total Dental Index (TDI), describing the inflammatory load caused by oral infections, and Panoramic Tomography Index (PTI), additionally describing the inflammatory load by oral infections and the total number of teeth, reflecting long-term inflammatory load [36,37,38] (Table 1).

### 2.2. Statistical Analysis

All statistical analyses were performed with the software package IBM SPSS for Macintosh (version 26.0, IBM Corp., Armonk, NY, USA). Categorical variables were cross-tabulated and analyzed with Pearson’s Chi-square test or Fisher’s exact test if expected values in cell frequencies were <5. For differences in continuous variables, Student’s *t*-test, Mann–Whitney U test, one-way analysis of variance, and Kruskal–Wallis H test were used. Pairwise comparisons were performed as post hoc analyses for Pearson’s Chi-square test using Z test and Dunn’s (1964) procedure for the Kruskal–Wallis H test, both with a Bonferroni correction for multiple comparisons. *p*-values < 0.05 were considered statistically significant throughout the study.

## 3. Results

### 3.1. Characteristics of OTSCC Patients

None of the patients needed to be excluded based on the exclusion criteria. Findings of, in total, 183 consecutive patients with OTSCC were included in the final study cohort. The age of the patients ranged from 23 to 91 years, and males (*n* = 102) were more numerous than females (*n* = 81). Anamnestic information was available for 133 patients about smoking status and 87 patients about alcohol consumption (Table 2).

### 3.2. Differences in Male and Female Gender

Alcohol consumption was more prevalent among males than females (*p* = 0.028). Additionally, smoking was more common among males; however, the difference was not statistically significant. OLP and/or LR (*p* < 0.001) and intraepithelial *Candida* hyphae (*p* = 0.005) were significantly associated with the female gender. Males had higher PTI values than females (IQR 0–4 vs. 0–2, *p* = 0.036), but no significant differences existed between the sexes in TDI or PIBI values or number of teeth (Table 3).

### 3.3. OPMDs Association with Smoking and Alcohol Use

There was a significant association of the studied OPMDs (oral leukoplakia, OLP, and lichenoid reaction) both with smoking (*p* = 0.040) and alcohol use (*p* = 0.005). Patients with OLP/LR but not with leukoplakia were less often smokers than patients with neither OLP/LR nor leukoplakia. Patients with OLP/lichenoid reaction but not with leukoplakia and patients with leukoplakia but no OLP/LR were associated with abstinence from alcohol usage (*p* = 0.005) (Table 4).

### 3.4. Oral Candida Hyphae Associated with No Alcohol Use

**Figure 1:**
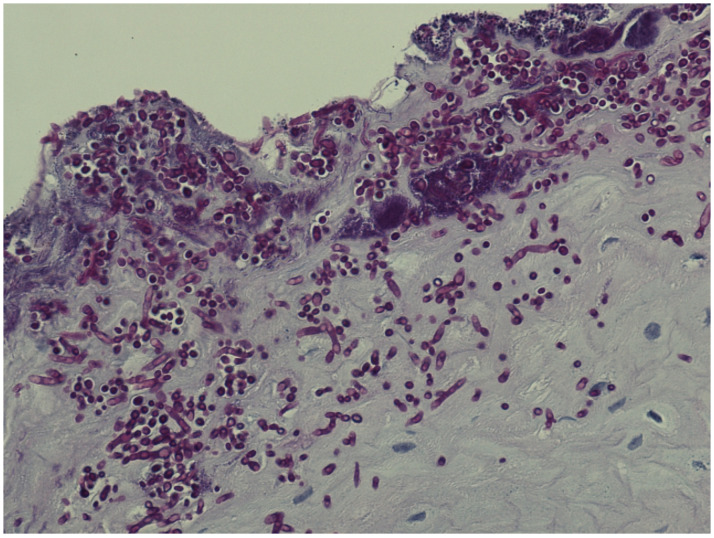
Plenty of *candida* hyphae in oral epithelium.

Patients with histologically verified oral *Candida* hyphae (Figure 1) were significantly older than patients without (*p* = 0.003), with a medium effect size (Hedges’ g = 0.544). Oral *Candida* hyphae were more often observed in patients with no alcohol usage (*p* = 0.012) (Table 5).

## 4. Discussion

The purpose of this study was to address the role of OPMDs and intraepithelial *Candida* hyphae, indicative of *Candida* infection, in OTSCC development and to evaluate the effect of smoking and oral infection disease burden on this process. To address the oral inflammatory burden of common oral infections, such as dental caries and periodontal disease, the number of teeth, PIBI, TDI, and PTI were utilized as described elsewhere [36,37,38].

In our study, females more often had a history of OLP/LR and oral *Candida* hyphae, and less often smoking or alcohol consumption. By contrast, leukoplakia was evenly distributed between the genders. The prevalence of autoimmune diseases or immunosuppressive medication did not show a difference between men and women.

Patients with OLP/LR and patients who developed cancer of the tongue had no significant association with underlying risk factors, such as smoking or alcohol use, while in the group of patients with no premalignant mucosal lesions smoking and alcohol use were more common. Presumably, the patients’ gender partly explains the associations between smoking and alcohol consumption, as females, non-smokers, and non-alcohol-using groups had more OLP/LR. The results underline the fact that OLP/LR obviously belong to the OPMD group of mucosal lesions.

The clinical or radiological oral health status indices PIBI and TDI were not associated with OPMDs or the *Candida* hyphae findings. However, males had higher PTI values than females, which is in accordance with the 2011 national survey on the oral health of Finnish citizens (Health 2011 Survey) [39].

We did not observe a significant association between oral *Candida* hyphae findings and OPMDs in OTSCC patients [26]. This might reflect the fact that OPMDs and *Candida* both take part in the malignant process independently.

Intraepithelial *Candida* hyphae were less common in patients with smoking or alcohol consumption, even though these are generally known to predispose to *Candida* [40]. The reason for this may be that in our study, oral *Candida* hyphae associated significantly with the female sex, and women were less often smokers and alcohol users. Therefore, the difference in prevalence of *Candida* hyphae may also be, at least in part, due to the gender. It might additionally reflect the fact that information about the smoking status or alcohol use may not always be registered properly in medical records. There is a potential risk of underreporting unhealthy habits to healthcare professionals. Moreover, smoking and alcohol consumption can alter the virulence factors of *Candida* species [30]. Future studies should investigate the impact of smoking and alcohol use on carcinogenesis in patients with OLP/LR and oral candidiasis.

The information on *Candida* species by culture was available only for very few patients and, therefore, the meaning of specific species for carcinogenesis could not be addressed in our study. Further prospective studies on the association of these factors and OTSCC are warranted. Additionally, the biotype, genotype, and virulence factors of *Candida* species in different forms of OLP and LR and whether they are relevant in OTSCC carcinogenesis should be studied.

Hyposalivation and poor oral or denture hygiene are well-known predisposing factors for oral candidiasis in addition to mucosal barrier abnormality, systemic immunosuppressive diseases and medications, antibiotics, and heavy smoking [13,14,41]. Due to the retrospective nature of our study, we did not obtain a comprehensive understanding of the frequency of dry mouth or the use of dentures in patients. Related to oral hygiene as a risk factor for carcinogenesis, *Candida* and polymicrobial oral biofilms have been reported to increase the expression of the cancer-associated inflammatory cytokines IL-6 and IL-8 in normal and cancer cells [32]. Further prospective studies should be conducted to compare the differences in oral and dental health in subjects with OPMD lesions with or without progression to cancer to clarify the role of oral infection burden in inflammatory mediators and related carcinogenesis.

Limitations of this study include the lack of a control group. Therefore, this study does not address the causality of findings to the development of OTSCC. Another limitation is the retrospective nature of the study because patient information in medical records is not always thoroughly and uniformly recorded. In addition, appropriate information for the differential diagnostics between OLP and LR was not available due to the retrospective nature of the research. The strengths of the study are that the patients are consecutive OTSCC patients, and the number of patients is sufficiently large for statistical analyses. Further studies should be performed with a larger OTSCC patient cohort in order to verify if the current findings are generalizable to a larger population. A prospective study would enable the comprehensive collection of *Candida* samples for species identification to provide more detailed insight into the mechanisms underlying the relation between *Candida* infection and OTSCC.

To conclude, OLP/LR and intraepithelial *Candida* hyphae were found to be associated with the female gender and less with typical OTSCC risk factors such as alcohol use and smoking. It is important to recognize that this group of patients is at risk for oral cancer development and to monitor these patients frequently enough to enable the early diagnosis of cancer.

## Figures and Tables

**Table 1:** Classification of oral inflammatory burden. Oral inflammatory burden can be assessed by recording different indices, such as the PIBI, TDI, and PTI, as shown in the next tables [16,24].

PIBI = Nmod PPD + 2 Nadv PPD		
Nmod = number of sites with moderate periodontal lesions (4 ± 5 mm)		
Nadv = number of sites with advanced periodontal lesions (≥6 mm)		
PPD = periodontal pocket depth		
Determination of Periodontal Inflammatory Burden (PIBI, 28 teeth).		
Determination of Total Dental Index (TDI, 32 teeth).		
Oral Disease		Score
Caries	No caries lesions	0
	1 ± 3 caries lesions	1
	4 ± 7 caries lesions or no teeth in maxilla or mandible	2
	8 caries or radix or no teeth	3
Periodontitis	4 ± 5 mm deep periodontal pocket	1
	6 mm deep periodontal pocket	2
	Pus in gingival pocket	3
Periapical lesions	1 periapical lesion or vertical bone pocket or both	1
	2 periapical lesions	2
	3 periapical lesions	3
Pericoronitis	Absent	0
	Present	1
Determination of Panoramic Tomography Index (PTI)		
PTI = Periapical lesions + lesions caused by tertiary caries + vertical bone pockets + radiolucent areas at the furcation + lesions caused by pericoronitis formed		

**Table 2:** Characteristics of study participants.

Characteristic	N
Total	183 (100.0)
Gender	
Female	81 (44.3)
Male	102 (55.7)
Age (years)	60.4 ± 13.68 (median 60.5)
Smoking	
Current	56 (30.6)
Former	25 (13.7)
No	52 (28.4)
Record unavailable	50 (27.3)
Cigarette pack years ^1^	40 (30–41)
Alcohol use	
Current	65 (35.5)
Former	1 (0.5)
No	21 (11.5)
Record unavailable	96 (52.5)
Weekly alcohol doses ^2^	6.5 (3–15)
Autoimmune disease (*n* = 174)	25 (13.7)
Immunosuppressive medication (*n* = 174)	20 (10.9)
Oral mucosal diseases	
Leukoplakia	49 (26.8)
Lichen planus and/or lichenoid reaction	48 (26.2)
*Candida* (any species)	39 (21.3)
Verrucous leukoplakia	5 (2.7)
Number of teeth (*n* = 159)	25 (21–28)
TDI (*n* = 60)	3 (1–4)
PTI (*n* = 155)	1 (0–3)
PIBI (*n* = 37)	1 (0–20)

Data presented as means ± standard deviations or medians (and interquartile ranges) for continuous variables and as frequencies (and percentages) for categorical variables. ^1^ Record available for 41 current or former smokers. ^2^ Record available for 22 patients with current/regular alcohol consumption. Abbreviations: TDI, Total dental index; PTI, Panoramic tomography index; PIBI, Periodontal inflammatory burden index.

**Table 3:** Study variables stratified by gender.

Characteristic	Females (*n* = 81)	Males (*n* = 102)	*p-*Value
Age (years)	62.4 ± 13.23	58.8 ± 13.89	0.080
23—60	34 (42.0)	57 (56.4)	0.053
61—91	47 (58.0)	44 (43.6)	
Smoking (*n* = 133)			0.060
Current	17 (34.0)	39 (47.0)	
Former	7 (14.0)	18 (21.7)	
No	26 (52.0)	26 (31.3)	
Alcohol use (*n* = 87)			0.028 *
Yes (current/former)	23 (63.9)	43 (84.3)	
No	13 (36.1)	8 (15.7)	
Autoimmune disease (*n* = 174)	15 (19.7)	10 (10.2)	0.075
Immunosuppressive medication (*n* = 174)	10 (13.3)	10 (10.1)	0.508
Oral mucosal diseases			
Leukoplakia	24 (29.6)	25 (24.5)	0.437
Lichen planus/lichenoid reaction	32 (39.5)	16 (15.7)	<0.001 ***
*Candida*	25 (30.9)	14 (13.7)	0.005 **
Number of teeth (*n* = 159)	25 (21–28)	25 (20–28)	0.484
TDI (*n* = 60)	3 (0–3)	3 (1–5)	0.248
PTI (*n* = 155)	0 (0–2)	1 (0–4)	0.036 *
PIBI (*n* = 37)	0 (0–9)	2 (0–22)	0.071

Data presented as means ± standard deviations or medians (and interquartile ranges) for continuous variables and as frequencies (and percentages) for categorical variables. Age was compared with *t* test and other continuous variables with Mann–Whitney *U* test. Categorical variables were analyzed with Pearson’s Chi-square test. * Statistically significant (*p* < 0.050). ** Statistically significant (*p* < 0.010). *** Statistically significant (*p* < 0.001). Abbreviations: TDI, Total dental index; PTI, Panoramic tomography index; PIBI, Periodontal inflammatory burden index.

**Table 4:** Study variables stratified by presence of oral mucosal diseases.

Characteristic	Leukoplakia, No OLP/LR (*n* = 38)	OLP/LR, No Leukoplakia (*n* = 37)	Leukoplakia and OLP/LR (*n* = 11)	Neither Leukoplakia Nor OLP/LR (*n* = 97)	*p-*Value
Gender					0.003 **
Female	16 (42.1)	24 (64.9)	8 (72.7)	33 (34.0)	
Male	22 (35.1)	13 (35.1)	3 (27.3)	64 (66.0)	
Age (years)	58.5 ± 13.71	63.4 ± 12.16	62.6 ± 12.10	59.8 ± 14.36	0.395
23–60	21 (55.3)	17 (45.9)	4 (36.4)	49 (51.0)	0.677
61–91	17 (44.7)	20 (54.1)	7 (63.6)	47 (49.0)	
Smoking (*n* = 133)					0.040 *
Current	10 (32.3)	4 (17.4)	3 (75.0)	39 (52.0)	
Former	6 (19.4)	5 (21.7)	0	14 (18.7)	
No	15 (48.4)	14 (60.9)	1 (25.0)	22 (29.3)	
Alcohol use (*n* = 87)					0.005 **
Yes (current/former)	9 (52.9)	7 (53.8)	1 (50.0)	50 (87.7)	
No	8 (47.1)	6 (46.2)	1 (50.0)	7 (12.3)	
Autoimmune disease (*n* = 174)	5 (13.2)	7 (19.4)	2 (20.0)	11 (12.2)	0.707
Immunosuppressive medication (*n* = 174)	7 (14.9)	5 (13.5)	0	8 (8.9)	0.256
*Candida* by histopathologic examination	8 (21.1)	12 (32.4)	2 (18.2)	17 (17.5)	0.305
Number of teeth (*n* = 159)	27 (23–30)	24 (22–27)	26 (24–28)	25 (18—28)	0.894
TDI (*n* = 60)	1.5 (0–4)	2 (0–4)	3 (2–5)	3 (1—5)	0.579
PTI (*n* = 155)	0 (0–2)	1 (0–3)	1 (0–2)	1 (0—4)	0.234
PIBI (*n* = 37)	0 (0–2)	2 (0–29)	NA	1 (0—21)	0.565

Data presented as means ± standard deviations or medians (and interquartile ranges) for continuous variables and as frequencies (and percentages) for categorical variables. Age was compared with ANOVA and other continuous variables with Kruskal–Wallis *H* test. Categorical variables were analyzed with Pearson’s Chi-square test. * Statistically significant (*p* < 0.050). ** Statistically significant (*p* < 0.010). Abbreviations: OLP/LR, oral lichen planus and/or lichenoid reaction; TDI, Total dental index; PTI, Panoramic tomography index; PIBI, Periodontal inflammatory burden index.

**Table 5:** Study variables stratified by presence of *Candida* by histopathologic examination (any species).

Characteristic	*Candida* Pos. (*n* = 39)	*Candida* Neg. (*n* = 144)	*p-*Value
Gender			0.005 **
Female	25 (64.1)	56 (38.9)	
Male	14 (35.9)	88 (61.1)	
Age (years)	66.1 ± 11.71	58.8 ± 13.80	0.003 **
23–60	15 (38.5)	76 (53.1)	0.104
61–91	24 (61.5)	67 (46.9)	
Smoking (*n* = 133)			0.063
Current	6 (28.6)	50 (44.6)	
Former	2 (9.5)	23 (20.5)	
No	13 (61.9)	39 (4.8)	
Alcohol use (*n* = 87)			0.012 *
Yes (current/former)	6 (46.2)	60 (81.1)	
No	7 (53.8)	14 (18.9)	
Autoimmune disease (*n* = 174)	7 (18.9)	18 (13.1)	0.374
Immunosuppressive medication (*n* = 174)	6 (16.7)	14 (10.1)	0.376
Number of teeth (*n* = 159)	24 (19–26)	26 (21–28)	0.113
TDI (*n* = 60)	3 (1–5)	3 (1–4)	0.729
PTI (*n* = 155)	2 (0–4)	1 (0–2)	0.073
PIBI (*n* = 37)	0 (0–21)	2 (0–20)	0.481

Data presented as means ± standard deviations or medians (and interquartile ranges) for continuous variables and as frequencies (and percentages) for categorical variables. Age was compared with *t* test and other continuous variables with Mann–Whitney *U* test. Categorical variables were analyzed with Pearson’s Chi-square test or Fisher’s exact test. * Statistically significant (*p* < 0.050). ** Statistically significant (*p* < 0.010). Abbreviations: TDI, Total dental index; PTI, Panoramic tomography index; PIBI, Periodontal inflammatory burden index.

## Data Availability

The data supporting the results reported here are available from the corresponding author upon request.
